# Transcription factors KLF15 and PPARδ cooperatively orchestrate genome-wide regulation of lipid metabolism in skeletal muscle

**DOI:** 10.1016/j.jbc.2022.101926

**Published:** 2022-04-09

**Authors:** Liyan Fan, David R. Sweet, Erica K. Fan, Domenick A. Prosdocimo, Annmarie Madera, Zhen Jiang, Roshan Padmanabhan, Saptarsi M. Haldar, Vinesh Vinayachandran, Mukesh K. Jain

**Affiliations:** 1 Case Cardiovascular Research Institute, Case Western Reserve University, and Harrington Heart and Vascular Institute, University Hospitals Cleveland Medical Center, Cleveland, Ohio, USA; 2 Department of Pathology, Case Western Reserve University, Cleveland, Ohio, USA; 3 University of Pittsburgh School of Medicine, Department of Physical Medicine and Rehabilitation, Pittsburgh, Pennsylvania, USA; 4 The Webb Law Firm, Pittsburgh, Pennsylvania, USA; 5 Gladstone Institute of Cardiovascular Disease, San Francisco, California, USA; 6 Department of Medicine, Division of Cardiology, University of California San Francisco School of Medicine, San Francisco, California, USA

**Keywords:** Krüppel-like factor (KLF), metabolism, energy metabolism, skeletal muscle, transcription, transcription factors, peroxisome proliferator–activated receptors, nuclear receptors, KLF15, Krüppel-like factor 15, KO, knockout, NR, nuclear receptor, PPAR, peroxisome proliferator–activated receptor, PPRE, peroxisome proliferator response element

## Abstract

Skeletal muscle dynamically regulates systemic nutrient homeostasis through transcriptional adaptations to physiological cues. In response to changes in the metabolic environment (*e.g.*, alterations in circulating glucose or lipid levels), networks of transcription factors and coregulators are recruited to specific genomic loci to fine-tune homeostatic gene regulation. Elucidating these mechanisms is of particular interest as these gene regulatory pathways can serve as potential targets to treat metabolic disease. The zinc-finger transcription factor Krüppel-like factor 15 (KLF15) is a critical regulator of metabolic homeostasis; however, its genome-wide distribution in skeletal muscle has not been previously identified. Here, we characterize the KLF15 cistrome *in vivo* in skeletal muscle and find that the majority of KLF15 binding is localized to distal intergenic regions and associated with genes related to circadian rhythmicity and lipid metabolism. We also identify critical interdependence between KLF15 and the nuclear receptor PPARδ in the regulation of lipid metabolic gene programs. We further demonstrate that KLF15 and PPARδ colocalize genome-wide, physically interact, and are dependent on one another to exert their transcriptional effects on target genes. These findings reveal that skeletal muscle KLF15 plays a critical role in metabolic adaptation through its direct actions on target genes and interactions with other nodal transcription factors such as PPARδ.

Skeletal muscle has robust capacity to remodel its metabolic machinery to adapt to changes in physical activity and nutrient availability (1, 2, 3). Numerous studies in animals and humans have demonstrated that skeletal muscle’s ability to efficiently and appropriately metabolize sugars and fats is a key determinant of systemic metabolic function and exercise capacity (4). Furthermore, derangements in muscle metabolism have been shown to greatly contribute to the pathology of metabolic disorders such as obesity, type II diabetes mellitus, and nonalcoholic fatty liver disease (4, 5). These observations underscore the importance of understanding the molecular mechanisms underlying skeletal muscle adaptation to metabolic cues.

The transcriptional control of muscle metabolism has garnered much attention over the last few decades, fueled by the discovery of links between the activation of different transcriptional networks by different metabolic states and challenges (6, 7). Krüppel-like factor (KLF) 15, a member of the family of zinc-finger transcription factors, has been identified as a major regulator and effector of metabolic processes extending from nutrient acquisition to utilization (8). KLF15’s expression in metabolically active tissues (*e.g.*, liver, skeletal muscle, heart), its role in the metabolism of all three major nutrient classes, and the nature of its circadian oscillations define it as a critical player in regulating metabolic networks (9, 10, 11, 12, 13). Metabolic characterization of *Klf15* knockout (KO) mice, and more recently of muscle-specific *Klf15* KO mice (K15-SKO), demonstrates pronounced defects in lipid utilization, decreased exercise capacity, and susceptibility to diet-induced obesity (14, 15).

While the transcriptome driven by KLF15 has been well characterized across different tissue beds, we now turn our attention to the KLF15 cistrome and its interactions with other important metabolic regulators to fine-tune transcriptional activity. Of particular interest is the nuclear receptor (NR) superfamily of transcription factors, whose ligand-regulated function allows tight coupling of changes in the cellular environment with changes in transcription. The peroxisome proliferator–activated receptors (PPARs) are an important subfamily of ligand-activated NRs whose functions include the transcriptional regulation of cellular development and differentiation, nutrient homeostasis, and metabolism. In mammals, three members of this family have been identified: PPARα, PPARβ/δ, and PPARγ. All three isoforms are activated by natural fatty acids and their derivatives, whose sources include diet, *de novo* lipogenesis, and lipolysis, as well as synthetic lipophilic acids (16). The PPARs are of significant interest given their potential as therapeutic targets for a range of diseases, both metabolic and nonmetabolic (17). Small-molecule PPARα agonists, that is, fibrates, are used in the treatment of hypertriglyceridemia (18) and thiazolidinedione-class PPARγ agonists are used in managing type II diabetes mellitus (19).

While there are currently no PPARδ agonists in clinical use, promising studies in animals have demonstrated that activating PPARδ has beneficial effects on obesity, hypercholesterolemia, insulin resistance, and exercise endurance (20, 21). Mice systemically deficient in *Ppard* have limited viability due to defects in placentation, adipose mass, myelination, and inflammation (22, 23, 24). PPARδ is the dominant isoform in adult skeletal muscle, and mice with skeletal muscle–restricted PPARδ deficiency share many of the same characteristics as mice with skeletal muscle–specific KLF15 deficiency: both strains suffer from abnormalities in exercise capacity, lipid utilization, and oxidative metabolism and are susceptible to diet-induced obesity (25, 26, 27). Such similarities suggest potentially overlapping or synergistic roles of these important transcription factors.

Here, for the first time, we characterize the KLF15 cistrome *in vivo* in skeletal muscle, shedding light on the mechanism by which KLF15 controls transcription of target genes. By using PPARδ as an example for KLF15 interaction with other metabolic regulators, we demonstrate that KLF15 and PPARδ colocalize and interact to impact the transcription of target genes. Furthermore, *Klf15* deletion significantly altered PPARδ binding patterns and attenuated PPARδ transcriptional activity at critical lipid metabolism genes both at baseline and in response to pharmacological PPARδ agonism. These findings demonstrate KLF15’s role as a core component in metabolic transcriptional networks through its actions of facilitating the binding and activity of other transcription factors, thus fine-tuning the responses to different nutrient states and challenges that are requisite for homeostasis.

## Results

### *In vivo* assessment of KLF15 cistrome in skeletal muscle

Investigations into the KLF15-dependent transcriptome across multiple organs have demonstrated that KLF15 significantly impacts lipid, carbohydrate, and amino acid metabolic pathways in response to fluctuating nutrient challenges. The extent and manner in which KLF15 exerts its transcriptional effect through its occupancy at certain targets (*i.e.*, the KLF15 cistrome), however, remains to be defined in skeletal muscle. Recently, a mouse model harboring a *Klf15* “knock-in” allele containing a 3xFLAG epitope tag fused to the C-terminus of KLF15 has been developed and characterized, allowing for robust detection of endogenous KLF15 protein *in vivo* and ChIP-seq in the adult mouse liver (28). Using this mouse, we performed KLF15 ChIP-mentation, a technique that combines chromatin immunoprecipitation with tagmentation (sequencing library preparation by Tn5 transposase), in skeletal muscle tissue of healthy adult mice to define direct targets of KLF15.

**Figure 1:**
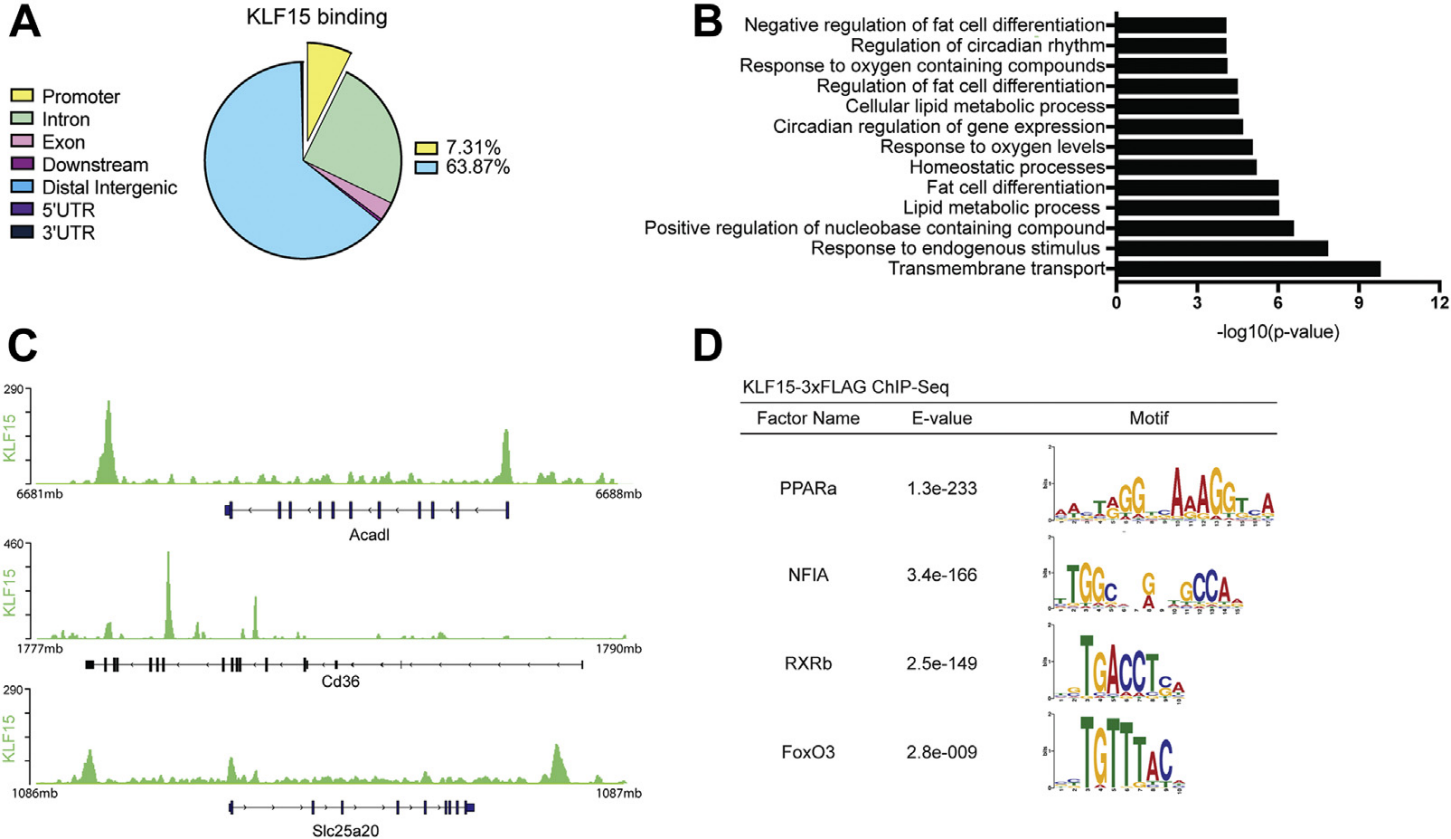
***In vivo* assessment of the KLF15 cistrome in skeletal muscle.***A*, distribution of KLF15 binding at different genomic locations. *B*, pathway analysis of enriched peaks from KLF15-3xFLAG ChIP-Seq. *C*, representative tracks from KLF15-3xFLAG ChIP-Seq showing KLF15 binding near metabolic genes. *D*, examples from motif analysis ± 200 bp from the center of significantly enriched peaks showing motifs for several important metabolic transcription factors. KLF15, Krüppel-like factor 15; PPAR, peroxisome proliferator–activated receptor.

Nearly 5000 KLF15-binding sites were determined, with 7.31% located within gene promoter regions and 63.78% located in distal intergenic regions (Fig. 1*A*). Gene ontology analysis on proximal targets of KLF15 peaks revealed enrichment of similar pathways seen in previous transcriptomic investigations of muscle KLF15; that is, KLF15 demonstrates increased binding at genes related to circadian rhythmicity and lipid metabolism, the transcription of which are attenuated in the absence of KLF15 (Fig. 1, *B* and *C*) (11, 15). Motif analysis of 200 bp upstream and downstream of KLF15-binding sites identified several motifs associated with critical metabolic transcription factors (*i.e.*, PPARa, RXRb, FoxO3) (Fig. 1*D*). This colocalization of binding motifs suggested a possible cooperative role between KLF15 and other transcription factors that bind at these locations. Notably, the peroxisome proliferator response element (PPRE), which binds the PPAR family of NRs, was among one of the most highly enriched motifs found near KLF15-binding sites (Fig. 1*D*).

### KLF15 and PPARδ demonstrate colocalization

Transcriptional activation occurs in the context of networks of transcription factors and coregulators that cooperate to respond to stimuli. To investigate how KLF15 might interact with other transcription factors within metabolic gene-regulatory networks, we chose to focus on the relationship between KLF15 and PPARδ. These factors share significant overlap in molecular function, are transcriptionally induced by similar physiological stimuli (*e.g*., fasting, exercise), and manifest similar phenotypes when targeted in mice (14, 21, 26, 29). When considered alongside our finding that the PPRE is significantly enriched near KLF15-binding sites, these observations suggest that KLF15 and PPARδ interact and operate on similar transcriptional programs.

**Figure 2:**
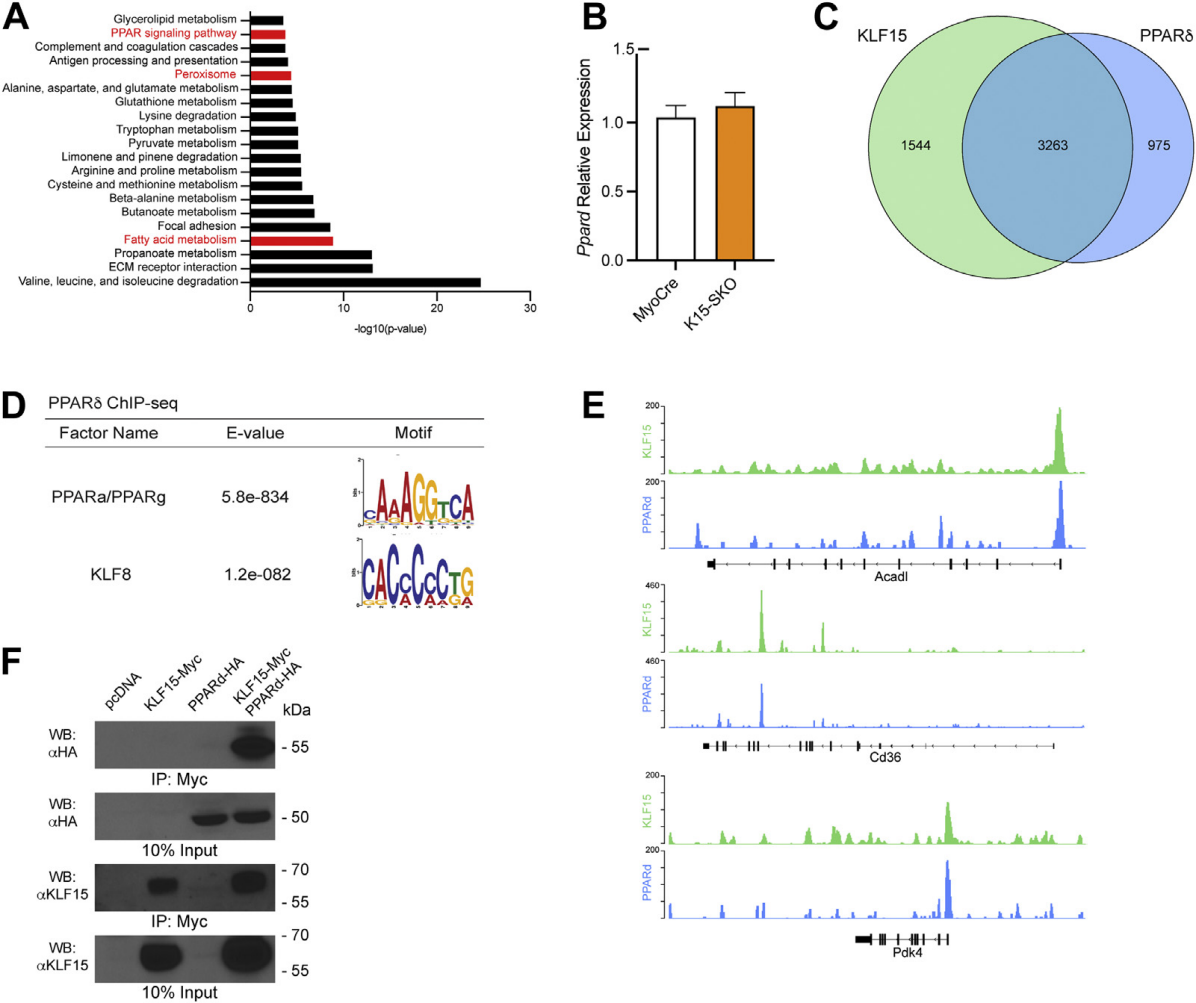
**KLF15 and PPAR**δ **demonstrate colocalization in skeletal muscle.***A*, top 20 most significantly enriched KEGG pathways from gene-set enrichment analysis of DEGs from MyoCre and K15-SKO muscle RNA-sequencing data. *B*, Ppard mRNA expression in MyoCre vs K15-SKO (n=5–6). *C*, Venn diagram demonstrating overlap of KLF15 binding and PPARδ binding from KLF15-3xFLAG ChIP-seq and PPARδ ChIP-seq, respectively. *D*, motif analysis of significantly enriched peaks from PPARδ ChIP-seq revealing both PPAR and KLF motifs. *E*, representative tracks demonstrating similar binding sites for KLF15 and PPARδ. *F*, overexpression of KLF15 and PPARδ in HEK-293 cells and co-IP demonstrating protein–protein interaction. co-IP, co-immunoprecipitation; KEGG, Kyoto Encyclopedia of Genes and Genomes; KLF15, Krüppel-like factor 15; PPAR, peroxisome proliferator–activated receptor.

Pathway analysis of the downregulated genes identified from RNA sequencing of K15-SKO muscle revealed significant enrichment for PPAR signaling pathway, peroxisome, and fatty acid metabolism (Fig. 2*A*). Importantly, *Ppard* mRNA expression is not affected in K15-SKO (Fig. 2*B*), suggesting that the presence of KLF15 may facilitate PPARδ-mediated transcription. PPARδ ChIP-seq corroborates these findings as significant overlap of PPARδ and KLF15 binding occurs in skeletal muscle. Indeed, of the 4238 PPARδ sites identified, 3263 (56.4%) were shared by KLF15 binding (Fig. 2, *B* and *C*). Motif analysis of 200 bp upstream and downstream of significantly enriched peaks in the PPARδ ChIP-seq revealed the presence of the PPRE consensus sequence, as well as the KLF motif (Fig. 2*D*), suggesting that PPAR NRs and KLFs bind in close proximity to one another and can coregulate gene transcription. We observed significant binding of both factors at a number of important metabolic genes (*e.g.*, *Acadl*, *Cd36*, and *Pdk4*) (Fig. 2*E*), strengthening previous evidence of these factors as critical metabolic regulators. Furthermore, co-immunoprecipitation studies using heterologous expression of epitope-tagged constructs demonstrated that KLF15 and PPARδ can form a complex (Fig. 2*F*). Altogether, these findings indicate that KLF15 and PPARδ colocalize across the genome of adult skeletal muscle and suggest that both transcription factors may function in an interdependent manner to coregulate a critical subset of target genes.

### KLF15 orchestrates PPARδ binding

**Figure 3:**
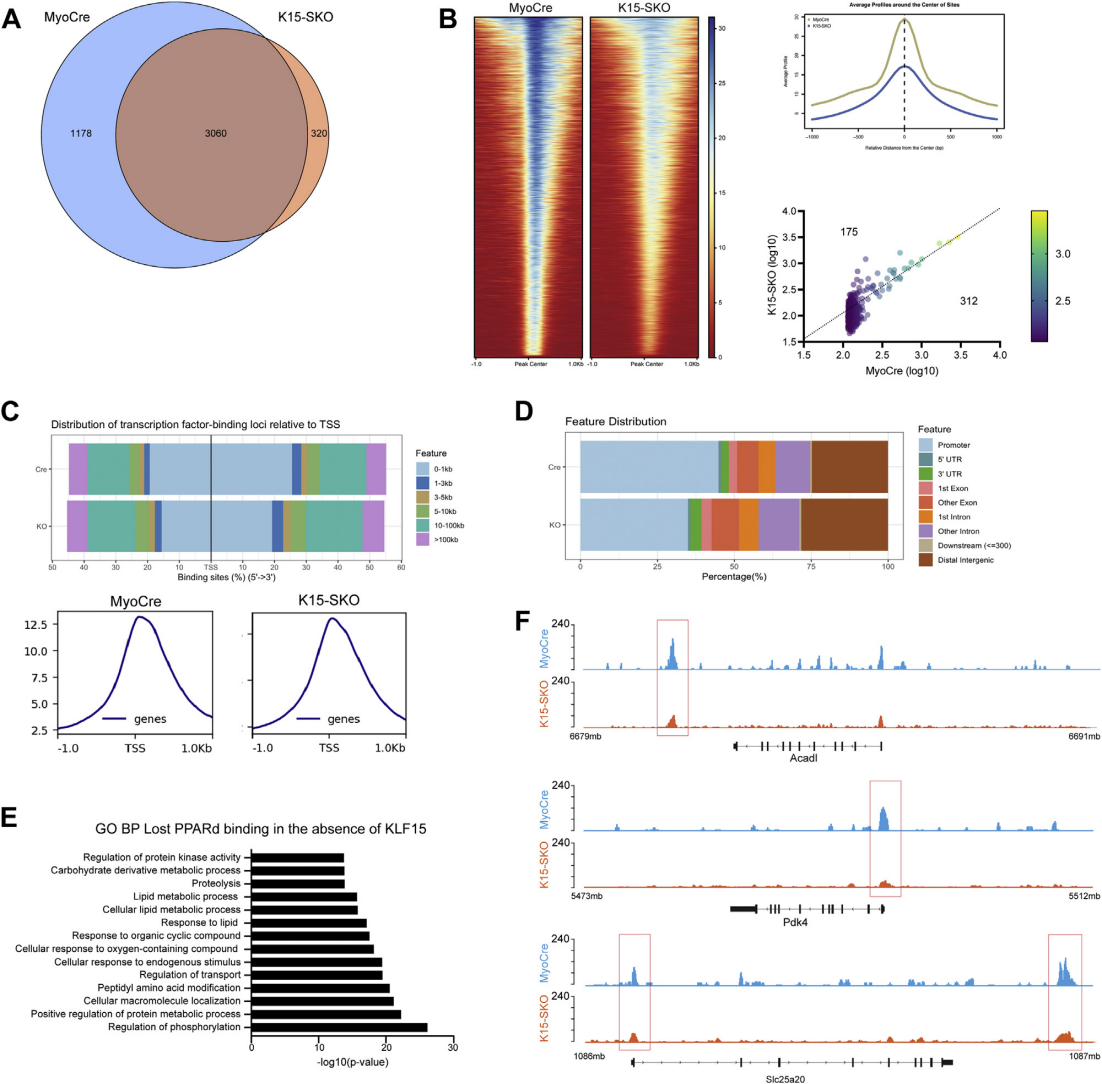
**KLF15 orchestrates PPARδ binding in skeletal muscle.***A*, Venn diagram demonstrating the overlap of PPARδ sites from PPARδ ChIP-seq in MyoCre vs K15-SKO. *B*, Heatmap of PPARδ ChIP-seq centered around all PPARδ peaks, showing loss of PPARδ binding in K15-SKO at the same locations. Top right: average profile of PPARδ binding centered around peak centers in MyoCre and K15-SKO. Bottom right: comparison of log-transformed enrichment scores for top 5% of PPARδ peaks showing increased binding in MyoCre. *C*, distribution of PPARδ binding relative to TSSs in the presence and absence of KLF15. *D*, distribution of genomic locations at which PPARδ is bound in MyoCre vs K15-SKO. *E*, pathway analysis of genes associated with decreased PPARδ binding in K15-SKO PPARδ ChIP-seq demonstrating enrichment for lipid metabolism pathways. *F*, representative tracks demonstrating decreased PPARδ binding at metabolic genes when KLF15 is absent. KLF15, Krüppel-like factor 15; PPAR, peroxisome proliferator–activated receptor; TSS, transcription start site.

These observations prompted us to further investigate whether PPARδ localization and function were dependent on the presence of KLF15. We performed PPARδ ChIP-seq in K15-SKO muscle and found that 1178 PPARδ-binding sites (27.80% of all PPARδ binding sites) showed decreased PPARδ enrichment in the absence of KLF15 (Fig. 3*A*). PPARδ enrichment at 3060 PPARδ-binding sites remained unchanged. However, a comparison of binding intensity and degree of enrichment at all PPARδ-binding sites demonstrated an overall decrease in PPARδ binding in K15-SKO (Fig. 3*B*). Additionally, there was a change in PPARδ global distribution patterns in the absence of KLF15: PPARδ binding near transcription start sites was significantly decreased (Fig. 3*C*), and there was a shift away from promoter regions with a relative increased binding at distal intergenic regions (Fig. 3*D*). Interestingly, we observed decreased PPARδ binding at several important metabolic genes, especially those related to lipid metabolism (Fig. 3, *E* and *F*). These results demonstrate that KLF15 is required for PPARδ recruitment to specific genomic loci, suggesting that endogenous KLF15 coordinates PPARδ-mediated transcriptional responses.

### KLF15 is necessary for optimal PPARδ target induction

**Figure 4:**
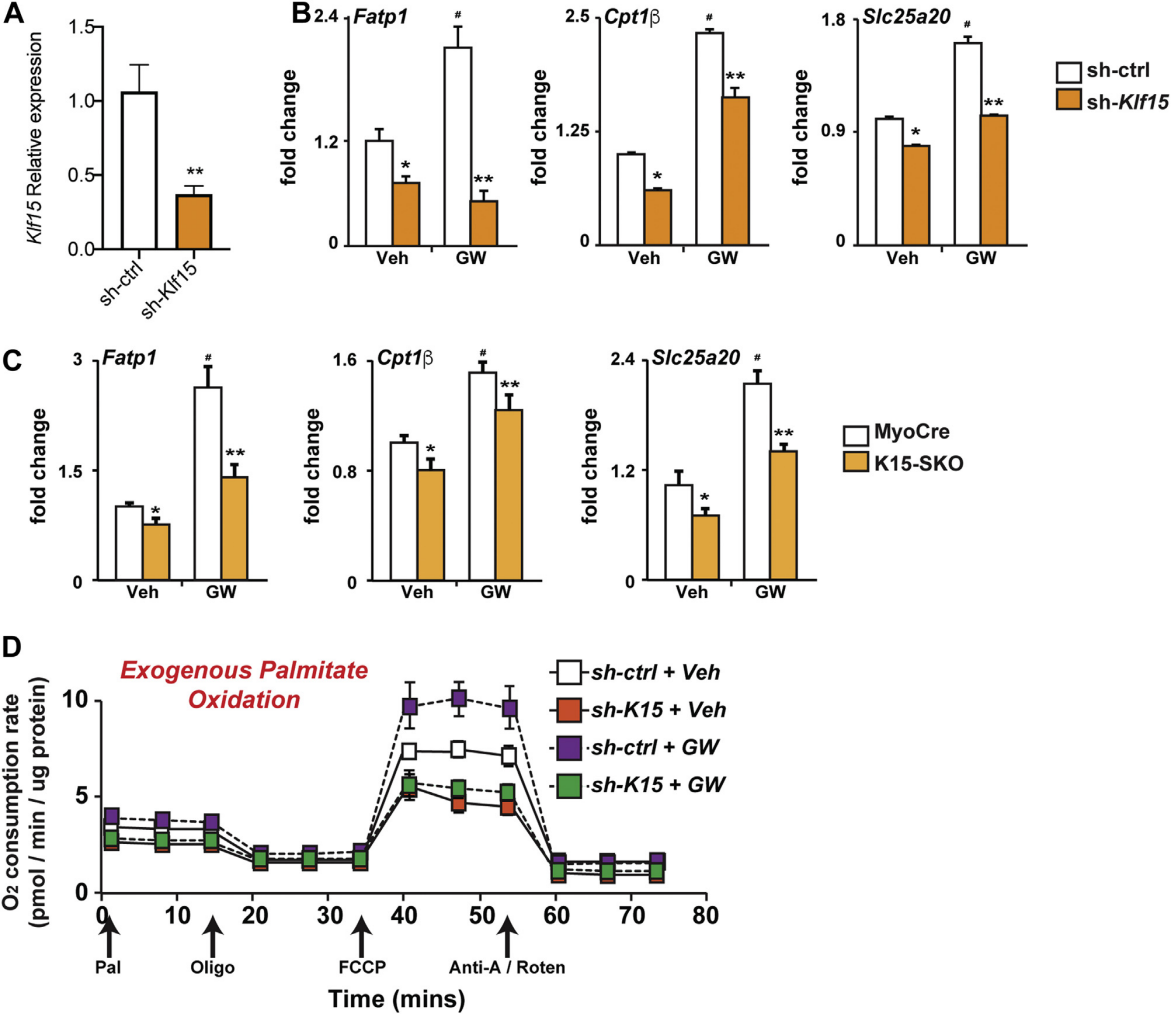
**KLF15 is necessary for optimal PPARδ control of lipid metabolism.***A*, relative *Klf15* expression in C2C12 cells following silencing with sh-Klf15 vs control (sh-ctrl). *B*, *Fatp1*, *Cpt1b*, and *Slc25a20* expression in differentiated C2C12 cells following acute silencing in the absence or presence of GW501516 (GW, 100 nM). *C*, *Fatp1*, *Cpt1b*, and *Slc25a20* expression in skeletal muscle of MyoCre *versus* K15-SKO mice following oral gavage of vehicle (Veh, DMSO) or GW (5 mg/kg body weight/day, 10 days). *D*, O_2_ consumption rate in differentiated C2C12 cells following acute Klf15 silencing in the absence or presence of GW501516 (100 nM, 24 h). Data represent mean ± SEM for n = 6. Comparisons between MyoCre and K15-SKO were performed using an unpaired, 2-tailed Student’s *t* test, ∗*p* < 0.05, ∗∗*p* < 0.01. KLF15, Krüppel-like factor 15; PPAR, peroxisome proliferator–activated receptor.

Because basal PPARδ localization appears to be dependent on KLF15, we sought to assess whether ligand-mediated agonism of PPARδ was similarly affected by the presence/absence of KLF15. To this end, we utilized the selective PPARδ agonist GW501516. GW501516 has been shown to impart a number of beneficial metabolic effects through its actions in increasing energy expenditure, inducing lipid metabolism genes, and increasing fatty acid oxidation (21, 26, 30, 31). Treatment of C2C12 cells, a myoblast cell line, with GW501516 (100 nM) augmented the expression of PPARδ target genes *Fatp1*, *Cpt1b*, and *Slc25a20*. Following silencing of KLF15, the induction of these genes by GW501516 was significantly attenuated (Fig. 4, *A* and *B*). This effect was confirmed *in vivo* by gavaging MyoCre and K15-SKO mice with GW501516 for 10 days (5 mg/kg body weight per day) followed by tissue collection. qPCR analysis showed that GW501516-stimulated gene induction of the PPARδ targets *Fatp1*, *Cpt1b*, and *Slc25a20* was attenuated in K15-SKO mice when compared to MyoCre controls (Fig. 4*C*). To assess whether KLF15 deficiency also affected PPARδ-dependent shifts in oxidative metabolism, we performed Seahorse profiling of cultured C2C12 cells in the setting of *Klf15* silencing and GW501516 stimulation. Similar to our gene expression analyses discussed earlier in the study, *Klf15* knockdown led to a decrease in basal palmitate oxidation and robustly inhibited the GW501516-stimulated augmentation of palmitate oxidation (Fig. 4*D*). Together, these data indicate that optimal PPARδ target gene induction and resultant increases in metabolic respiration are dependent on the presence of KLF15.

## Discussion

**Figure 5:**
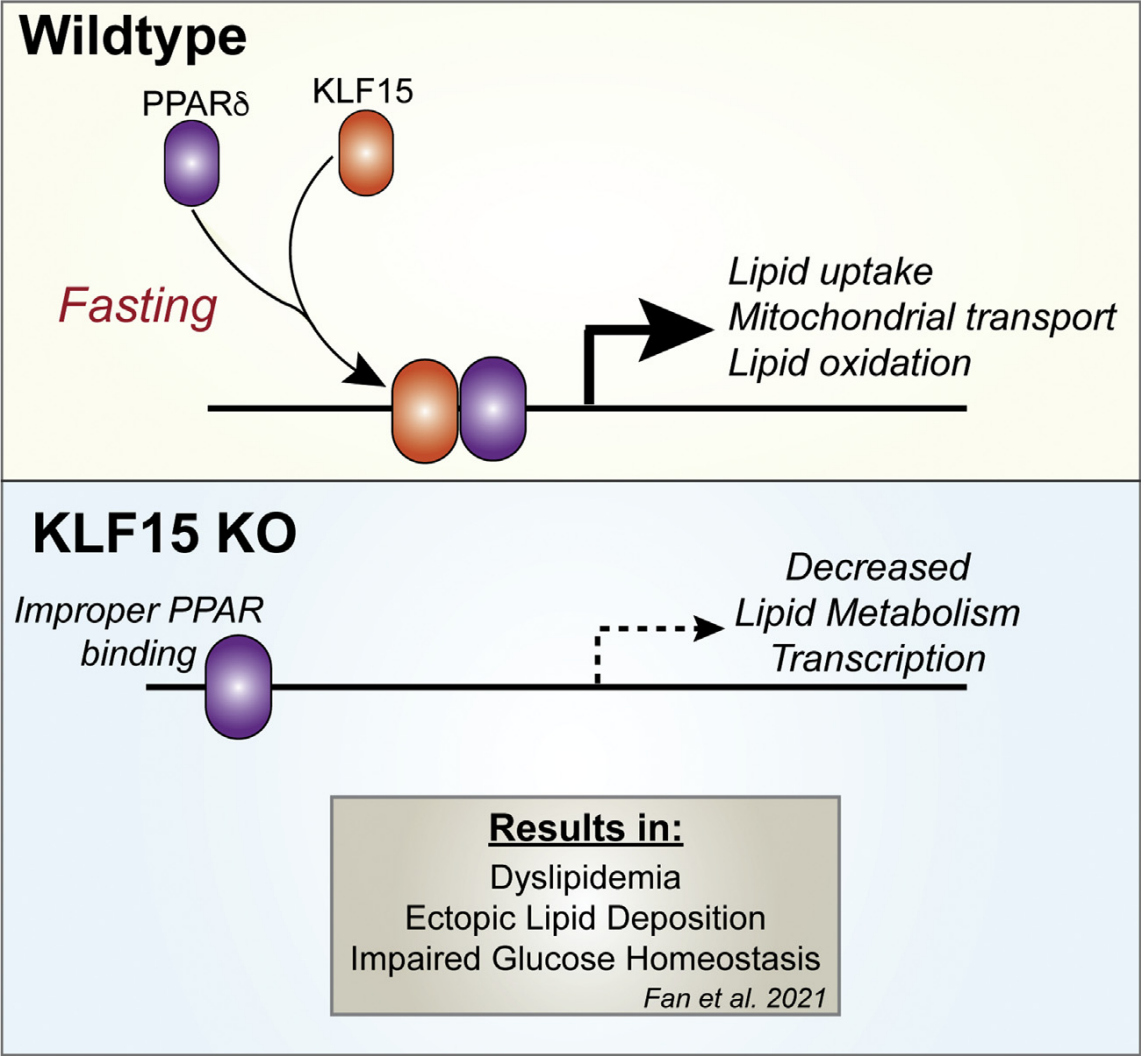
**Schematic representation of KLF15 and PPARδ interaction.** KLF15 and PPARδ colocalize and interact to regulate transcription of genes related to lipid uptake, mitochondrial transport, and lipid oxidation. In the absence of KLF15, PPARδ binding and subsequent transcriptional activity are altered. KLF15, Krüppel-like factor 15; PPAR, peroxisome proliferator–activated receptor.

Our findings provide new insights into KLF15 as a core component of systemic metabolism: through its own binding at target genes and through interactions with PPARδ, KLF15 reinforces transcription of critical metabolic programs in skeletal muscle (Fig. 5). To date, studies have largely focused on KLF15’s impact on the transcription of individual genes, with more recent studies surveying the KLF15 transcriptome; however, KLF15’s DNA binding pattern and genome-wide mechanisms of action in most tissues are still largely unknown. Here, using a recently developed mouse model with a “knock-in” allele that expresses a KLF15-3xFLAG fusion protein from the endogenous *Klf15* locus, we were able to characterize the KLF15 cistrome *in vivo* in skeletal muscle and demonstrate that genes and pathways previously shown to be downregulated in the absence of KLF15 are generally bound by KLF15 (11, 32, 33). A recent study using this mouse model in which KLF15 ChIP-seq was performed in the liver of adult mice showed that liver KLF15 predominantly exerts its actions through promoter binding and activation (32); interestingly, in skeletal muscle, we find that the majority of KLF15 binding occurs in the distal intergenic regions, similar to that of KLF1 binding in erythroid cells (34). This suggests that skeletal muscle KLF15 may participate in mechanisms of action in addition to classic promoter-based gene induction. There is a growing appreciation that gene expression is regulated by coordination between gene-proximal and gene-distant chromatin elements, with multiple transcription factors working in concert to dynamically establish cell state–specific gene expression programs. While the current work highlights how gene-proximal KLF15 binds and regulates its direct targets, additional work exploring a role for KLF15 in long-range gene regulation will further our understanding of context-dependent transcription in metabolic processes.

Due to the significant enrichment of PPREs near KLF15-binding sites and the similarities between the function of KLF15 and PPARδ in response to physiological cues, we turned to this family of NRs to illustrate KLF15’s potential action on proximally binding transcription factors. Our studies demonstrated that KLF15 and PPARδ colocalize on the skeletal muscle genome, can form a complex, and function in an interdependent manner to regulate lipid metabolism. In the absence of KLF15, PPARδ binding was lost near KLF15-binding sites, and PPARδ binding shifted away from promoter regions, suggesting that KLF15 helps recruit PPARδ to the correct transcriptional loci. However, given that KLF15 deficiency blunted downstream gene transcription rather than completely abolishing it, it is likely that other transcription factors or coactivators and corepressors act in tandem with the two under investigation here. In support of this, motif analysis of genomic regions flanking KLF15-binding sites demonstrated that other important metabolic factors (*e.g.*, FoxO and RXR) can bind in proximity to KLF15.

Previous work has shown that in the setting of fasting and nutrient depletion, related transcription factor cistromes are clustered together and dynamically affect one another in response to nutritional cues, leading to coactivation, repression, and a phenomenon termed “assisted loading” whereby one factor alters chromatin accessibility, thus allowing another factor to bind (35, 36). This mechanism allows the recruitment and coordination of large transcriptional networks in response to changes in the metabolic environment (35). The profound metabolic derangements observed in *Klf15*-deficient animals (*i.e.*, obesity, dyslipidemia, nonalcoholic fatty liver disease, etc.) suggest that KLF15 critically interacts with other transcription factors in these metabolic networks. Additionally, previous studies have shown that cardiac KLF15 binds and recruits the chromatin-remodeling enzyme p300 to promoters of genes controlling nutrient flux, thus possibly influencing chromatin accessibility for other transcription factors to bind (37). Indeed, it has been demonstrated that other members of the KLF family (*i.e.*, KLF4) facilitate the opening of chromatin that subsequently permits enhancer activation *via* the binding of additional transcription factors (38). In addition to the effects on coordinating PPARδ function observed in the current study, ongoing investigational efforts are now directed at assessing the extent to which KLF15 alters local chromatin state and enrichment of coregulatory transcriptional complexes. The results from these studies will deepen our understanding of how transcriptional responses to ever-changing metabolic states are fine-tuned and carried out to maintain organismal homeostasis.

## Experimental procedures

### Mice

The skeletal muscle–specific KLF15 deletion (K15-SKO) mouse model was generated by mating the Myogenin-Cre (MyoCre) mouse line to the Klf15^flox/flox^ mouse line, as previously described (15). The KLF15-3xFLAG mouse model was a generous gift from Saptarsi Haldar (Gladstone Institutes and University of California San Francisco) (32). In brief, this mouse model was generated by CRISPR-Cas9–based genome editing of mouse blastocysts whereby a cassette with a short linker sequence followed by a 3xFLAG tag was introduced immediately 5′ to the stop codon in exon 3 of the endogenous *Klf15* gene locus. Immediately following the final asparagine residue of mouse KLF15, this gene targeting strategy results in fusion of the following amino acid sequence to the C-terminus: GGGGADYKDHDGDYKDHDIDYKDDDDKGPV∗. All KLF15-3xFLAG mice are on a pure C57Bl/6 background. Detailed characterization of this mouse strain and the methods used to generate it can be found in the initial publication of this model (32). Age-matched, male, nonlittermate MyoCre mice were used as controls to K15-SKO. All mice are kept on a daily 12-h light–dark schedule, fed with tap water and standard chow ad libitum. All experiments involving animals were conducted under protocols approved by the Institutional Animal Care and Use Committee of Case Western Reserve University.

### RNA isolation and quantitative real-time PCR

Tissue samples were disrupted in PureZOL in a Tissue-lyzer (Qiagen) using stainless-steel beads (30 Hz for total 2 min). Total RNA was isolated using Bio-Rad Aurum Total RNA Fatty and Fibrous Tissue Kit using the manufacturer’s instructions and transcribed to complementary DNA using iScript (Bio-Rad). Quantitative real-time PCR was performed using Taqman method and appropriate probes from Roche Universal Probe Library System. Gene expression was normalized to cyclophilin B and compared using ΔΔCt method. All primers were efficiency tested and validated. Primers used in this study include the following: *Klf15* (forward: ACAGGCGAGAAGCCCTTT, reverse: CATCTGAGCGGGAAAACCT), *Cyclophilin B* (forward: TTCTTCATAACCACAGTCAAGACC, reverse: ACCTTCCGTACCACATCCAT), *Fatp1* (forward: GACAAGCTGGATCAGGCAAG, reverse: GAGGCCACAGAGGCTGTTC), *Cpt1b* (forward: GAGTGACTGGTGGGAAGAATATG, reverse: GCTGCTTGCACATTTGTGTT), *Slc25a20* (forward: TGAAGGCCCTGTTACACTCA, reverse: CCTCCAGAGAGTCAGCCATC)

### RNA-sequencing

The RNA sequencing data analyzed and discussed in this publication were from NCBI’s Gene Expression Omnibus dataset GSE160848. Significantly enriched Gene Ontology Biological Processes and Kyoto Encyclopedia of Genes and Genomes (KEGG) were generated by iDEP (39).

### ChIPmentation and analysis

Skeletal muscle was isolated from animals at 10 to 12 weeks of age and dissociated. Skeletal muscle cells were fixed using 1% formaldehyde, lysed, and frozen in 100 ul of SDS lysis buffer supplemented with 1× cOmplete EDTA-free protease inhibitor (Roche). For ChIP-Seq, for 100,000 to 150,000 cells, 5 uL of anti-FLAG (Cell Signaling Technologies, #8146) or anti-PPARb/d (F-10, Santa Cruz Biotechnology, sc-74517) antibodies were added to 25 uL of Protein A/Protein G-coupled Dynabeads (Bimake, B23202) in PBS with 0.5% BSA and incubated for 4 h at 4 degrees. Cells were sonicated for 12 cycles of 30 s on/30 s off on high power using a Bioruptor Pico (Diagenode). Triton X-100 (final concentration: 1%) and 50× cOmplete protease inhibitor (final concentration: 1×) were added to neutralize the SDS. Samples were incubated at RT for 10 min and 5% of the aliquots were saved for preparation of input controls. Antibody-coated Dynabeads were washed with PBS, mixed with cell lysate, and incubated overnight at 4 degrees with rotation. Immunoprecipitated chromatin was washed with 150 ul of low-salt buffer, high-salt buffer, and LiCl buffer, followed by two washes with TE buffer and two washes with ice-cold Tris/HCl (pH 8, 10 mM). For tagmentation, bead-bound chromatin was resuspended in 30 ul of tagmentation buffer. One microliter of transposase (Nextera, Illumina) was added, and samples were incubated at 37 degrees for 10 min, followed by two washes with low-salt buffer. For standard reverse cross-linking, chromatin complexes were diluted with 200 ul of ChIP elution buffer and 2 ul of 20 ug/ml proteinase K (Thermo Scientific), vortexed, and incubated overnight at 65 degrees. After reverse cross-linking, one ul of 20 ug/ml RNase (Sigma) was added and incubated at 37 degrees for 30 min. DNA purification was carried out using Qiagen MinElute PCR Purification Kit, and libraries were amplified and library cleanup was done using Agencourt AmPureXP beads (Beckman Coulter) at a ratio of 1:1. DNA concentrations in purified samples were measured using the Qubit dsRNA HS Kit (Invitrogen). Libraries were pooled, and single-end sequenced (50 cycles) using the NextSeq500 platform (Illumina). Initial quality check and adapter trimming was performed with Trim Galore (0.6.5) for ChIP-seq data (https://www.bioinformatics.babraham.ac.uk/publications.html). The ENCODE transcription factor ChIP-seq pipeline (40, 41) was used for alignment to the mm10 genome using bowtie2 (2.4.2) (42) and peak calling using MACS2 (2.2.7.1) (43). Deduplicated and uniquely mapped reads were used for peak calling analysis after excluding black-list regions following current ENCODE standardized guidelines. Called peaks for each biological replicate were processed using bedtools (2.30.0) (44) for intersections: overlapping peaks in at least two replicates were used for downstream analysis.

### GW501516 experiments

For *in vitro* experiments, C2C12 cells were differentiated for 6 days, infected with 1 uL of either sh-ctrl or sh-KLF15 for 1 day, and treated with either vehicle (DMSO) or GW501516 (100 nM) for 24 h. Cells were then harvested for RNA isolation and qPCR analysis. For *in vivo* experiments, mice were weighed and gavaged with either vehicle (DMSO in 5% carboxymethylcellulose) or GW501516 (in 5% carboxymethylcellulose) daily at 5 mg/kg body weight for 10 days. At the end of the experiment, animals were euthanized, and plasma and skeletal muscle were isolated for RNA isolation and qPCR analysis.

## Data availability

All data within the manuscript will be shared upon request (corresponding author). All ChIP-seq datasets have been deposited into NCBI’s Gene Expression Omnibus dataset GSE199547.

## Conflict of interest

S. M. H. is an executive, officer, and shareholder of Amgen and a scientific founder and shareholder of Tenaya Therapeutics. S. M. H. also serves on the Scientific Advisory Board for the German Centre for Cardiovascular Research of the German Ministry of Health (DZHK) on a voluntary and uncompensated basis. Z. J. is an employee of Biomarin. The other authors have declared that no conflict of interest exists.
